# Supplementary figures and images for: Clonal interference and genomic repair during strain coexistence in the gut
Source: PLoS Genet. 2025 Jul 7;21(7):e1011777. doi: 10.1371/journal.pgen.1011777 (PMC12251178; doi:10.1371/journal.pgen.1011777)

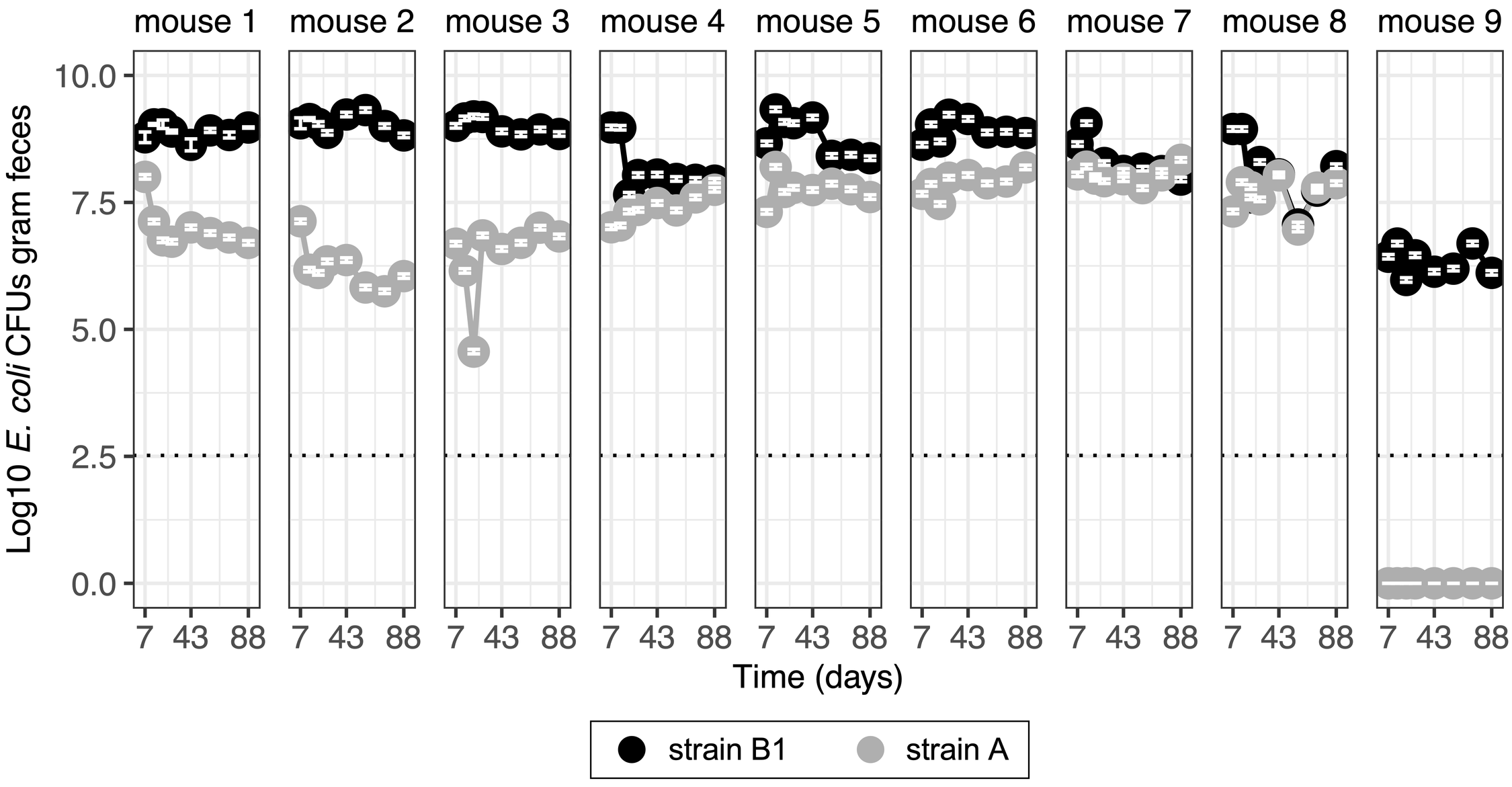

Supplement: S1 Fig — Gut co-colonization with both E. coli strains occurs in mice 1–8. In mouse 9 strain A failed to colonize. Error bars represent the Standard Error (2*SE). (TIF) [file pgen.1011777.s001.tif]

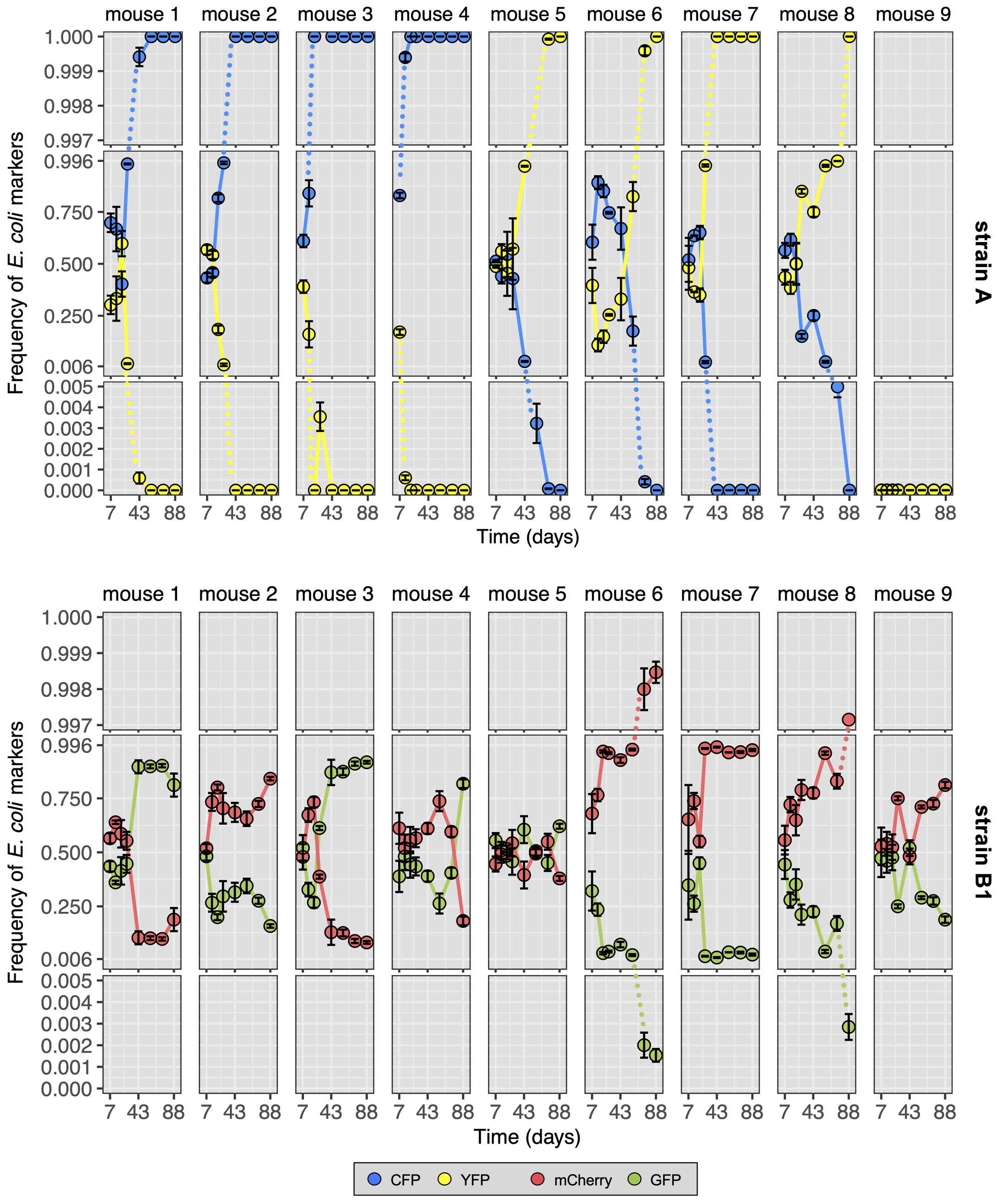

Supplement: S2 Fig — Gut co-colonization: frequency of the fluorescent markers in E. coli lineages of strain A (CFP, blue circles; YFP, yellow circles) and of strain B1 (mCherry, red circles; GFP, green circles) in the different mice (n = 9). Error bars represent the Standard Error (2*SE). In strain A, all mice exhibited fixation of one of the fluorescent markers, except for mouse 9, which was not successfully colonized. All the marker fixations can be attributed to a mutation reaching a frequency of 100%, except in the cases of mice 7 and 8. As no transfer of fluorescent markers or their linked resistance genes was observed, HGT events in other genomic regions must be invoked to explain neutral fluorescent marker fixation (S2 Table). In strain B1 there was maintenance of both fluorescence markers consistent with no selective sweeps observed (S3 Table). (TIF) [file pgen.1011777.s002.tif]

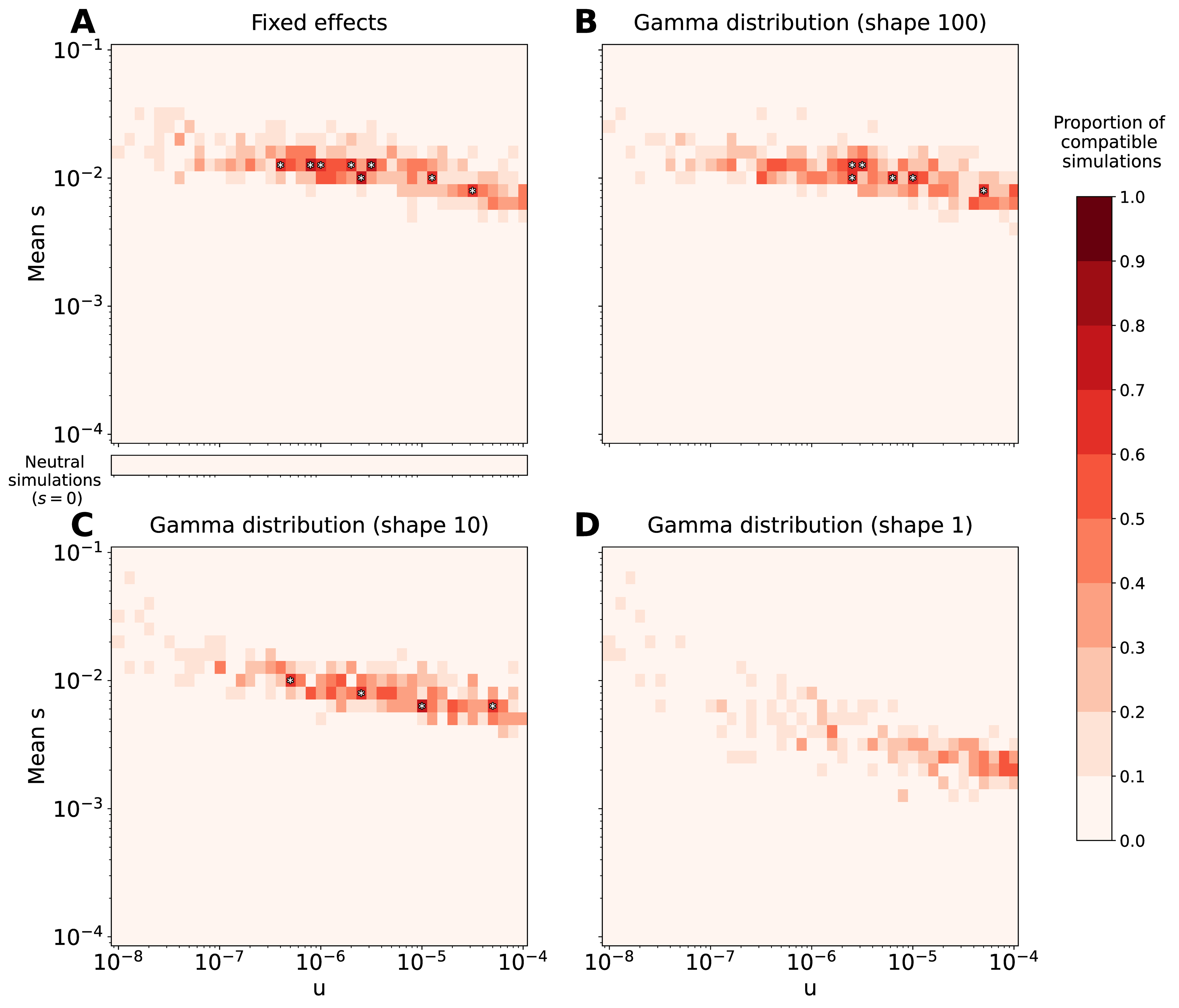

Supplement: S3 Fig — The heatmap shows the proportion of simulations compatible with the data of fluorescent marker abundances of each strain for each set of parameters (u and mean s). Data-compatibility is defined as maintaining only one marker in the strain with smaller population size (strain A in the experiment) and the two markers in the strain with larger population size (strain B1 in the experiment). Parameters were chosen as follows: total population N = 106 (which is a rough estimate of the number of bacteria in a typical mouse fecal sample), initial proportion of clones of strain A p = 0.1, number of generation g = 1600 (88 days with 18 generation per day as in the experiment). The mutation rate u was tested across a large range of values, as well as the mean selective effect s of newly arising beneficial mutations. Mutations were assumed to have a (A) fixed effect or to follow a (B-D) gamma distribution. Three different shapes of the gamma distribution were considered: (B) shape = 100, which gives similar results as those in a model that assumes that all mutations have the same s value (fixed effect); (C) shape = 10; and (D) shape = 1, which corresponds to an exponential distribution. 10 replicates were simulated for each parameter set. A black star indicates that a significant number of them was compatible with the experimental results of the 8 mice (Fisher exact test). Globally, these simulations show that these simple models could only explain the observed data under very restrictive sets of parameters (mainly on the mean s of the selective effect distribution and on its variance, which has to be very small). (TIF) [file pgen.1011777.s003.tif]

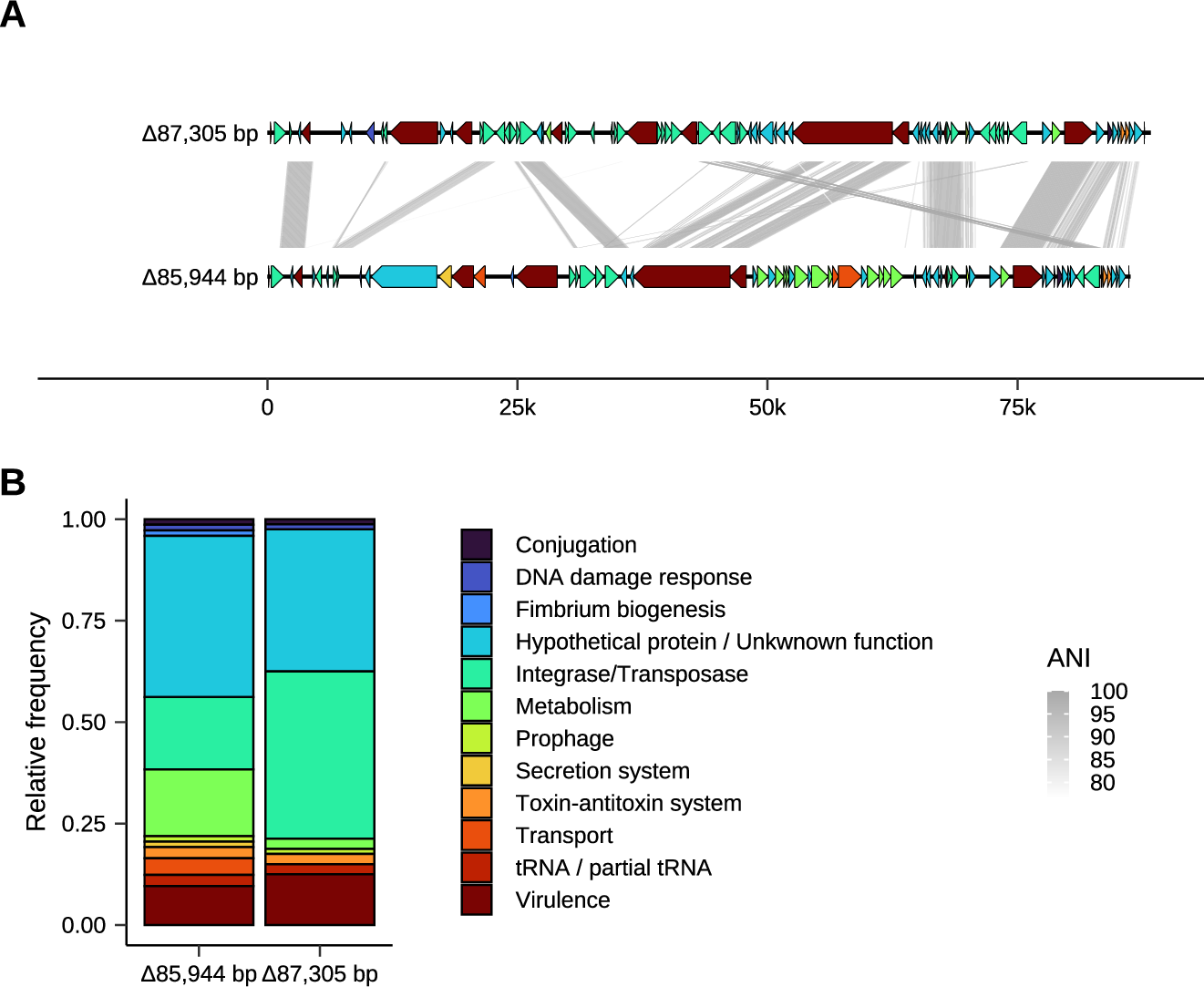

Supplement: S4 Fig — (A) Schematic representation of the genomic regions with homology between the two large deletions observed in strain B1. (B) Relative frequency of the predicted functions of the genes present in the two large deletions observed in strain B1. ANI, average nucleotide identity. Functional categories were obtained from the annotation of Prokka. (TIF) [file pgen.1011777.s004.tif]

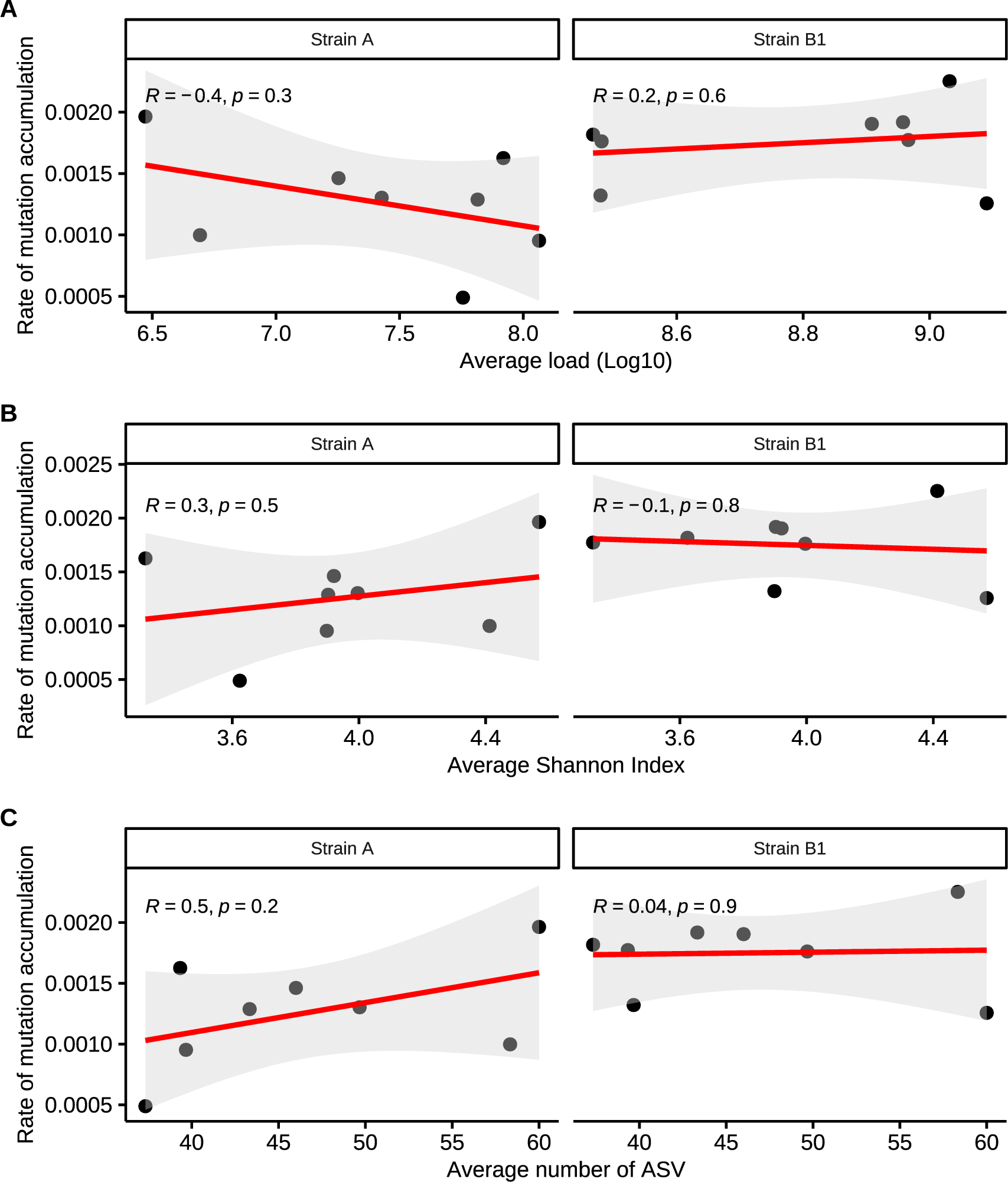

Supplement: S5 Fig — Correlation between the genomic rate of mutation accumulation for each mouse and the (A) Log10 of the average load (day 7–88), (B) average Shannon Index (day 13, 27, and 87), and (C) average number of amplicon sequence variants (ASV) (day 13, 27, and 87). For all panels, a Pearson’s correlation was performed. (TIF) [file pgen.1011777.s005.tif]

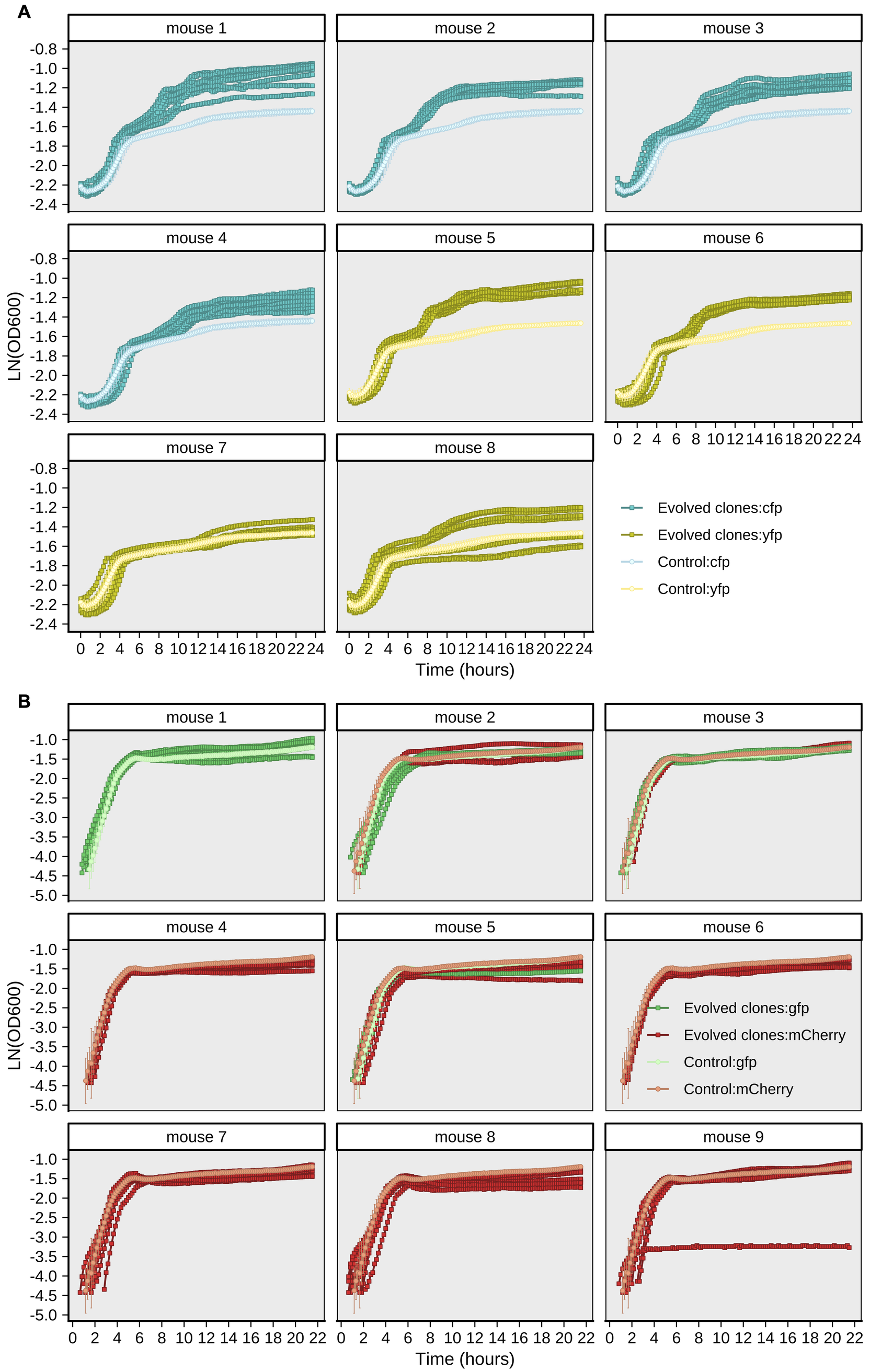

Supplement: S6 Fig — (A) Growth curves in media supplemented with mouse food of the ancestral (Control) and evolved clones of strain A sampled after three months of evolution inside the mouse gut. (B) Growth curves in media supplemented with mouse food of the ancestral (Control) and evolved clones of strain B1 sampled after 3 months of evolution inside the mouse gut. (TIF) [file pgen.1011777.s006.tif]

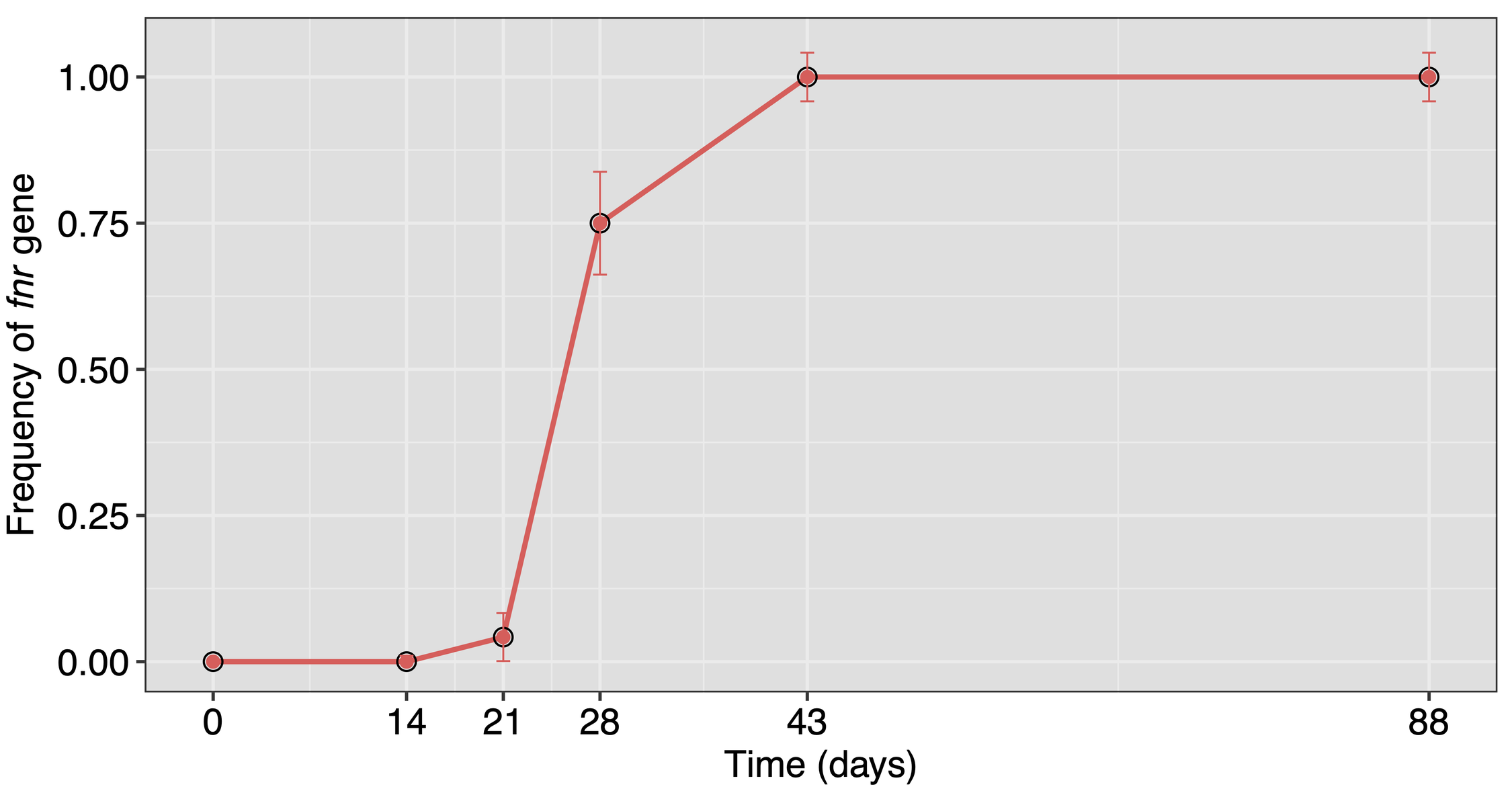

Supplement: S7 Fig — Dynamics of the spread of evolved clones which acquired a large lateral gene transfer event (which includes the fnr gene, typed here by colony PCR) located next to the prophage KingRac. (TIF) [file pgen.1011777.s007.tif]

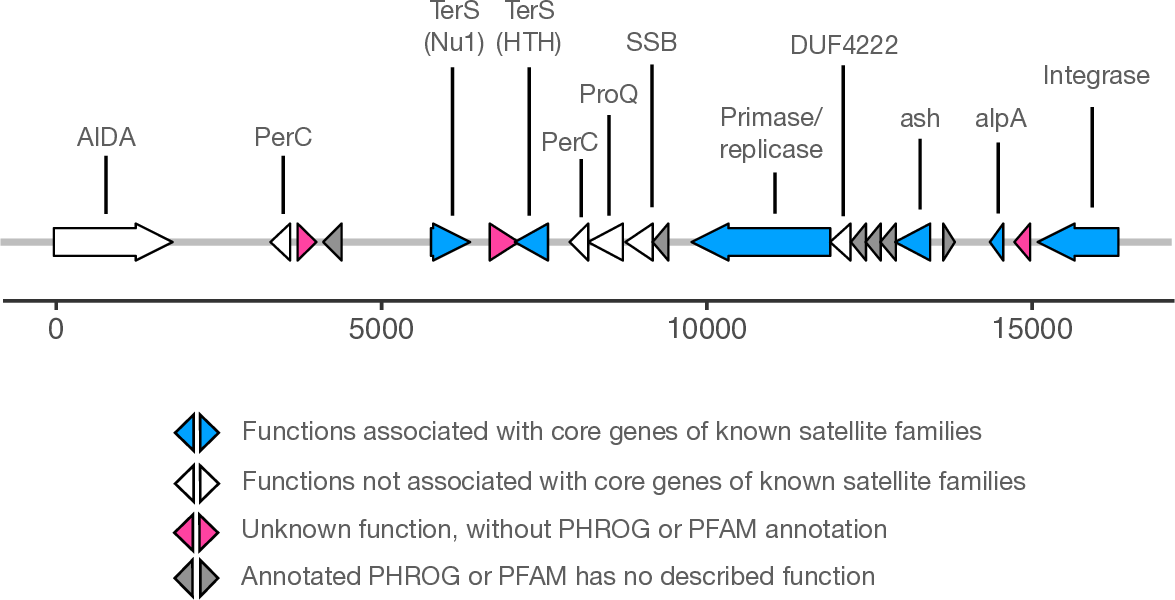

Supplement: S8 Fig — Scheme of the satellite genes and putative annotated functions. (TIF) [file pgen.1011777.s008.tif]
