# Supplementary material for: Clonal interference and genomic repair during strain coexistence in the gut
Source: PLoS Genet. 2025 Jul 7;21(7):e1011777. doi: 10.1371/journal.pgen.1011777 (PMC12251178; doi:10.1371/journal.pgen.1011777)
Supplement: Attachment — Submitted filename: Frazao_2024_review.pdf [file pgen.1011777.s021.pdf]

Clonal Interference and genomic repair during strain coexistence in the gut

Frazão et al., PLOS Genetics

Summary

This paper sets out to investigate the mode and tempo of evolution displayed by two co-colonizing, phylogenetically distinct strains of Escherichia coli in the mouse gut. Little is known about how evolution proceeds when two conspecific stains of the same bacterial species coexist in the same host gut. Frazão et al. find that the two strain populations can be distinguished according to their evolutionary dynamics: a mouse-associated strain (strain B1), which colonizes the mice at a significantly greater population size than the laboratory strain (strain A), shows a pattern consistent with clonal interference, whereby adaptive mutations sweep to intermediate frequency via partial sweeps (not necessarily "soft sweeps," as they are called in the paper, as elaborated on further below) and neutral variation was maintained. Contrastingly, evolution in the populations of a laboratory strain that colonized the mice is characterized by hard, complete sweeps in which adaptive mutations sweep to fixation, thereby eliminating neutral variation from the population. The study also finds evidence for extensive horizontal gene transfer (HGT) in which genetic material is transferred from the mouse-associated strain to the laboratory strain. Strikingly, one of these transfers restored genes important for gut colonization to the laboratory strain which that strain had previously lost during in vitro passaging. Finally, the study identifies what is likely the first documented instance of an in vivo transfer of a phage satellite—a mobile genetic element that exploits machinery of other prophages to transfer between cells.

Positives

This study addresses a significant gap in the literature by examining how bacterial strains evolve when co-colonizing the gut of the same host, as most previous research focuses on the evolutionary trajectories displayed by single strains. In pairing a fluorescence-based approach with pool sequencing, they are able to describe broadscale patterns in the evolutionary regimes operating on the two distinct strains (i.e., an evolutionary regime characterized by complete versus partial sweeps, respectively) as

well as the specific loci that are under selection and responsible for producing these broad-scale patterns. Altogether, this narrative they present is interesting and thoroughly told.

Moreover, the authors are able to offer convincing evidence that the evolutionary changes they document are likely adaptive, including by detecting widespread instances of parallelism between technical replicates of the same strain and evolutionary convergence between strains. It is quite an interesting finding that although the distinct strain populations show overlapping targets of adaptation, the manner in which they evolve is strain-specific—characterised by either partial or hard/complete sweeps, depending on the strain.

Finally, the authors do their due diligence in functionally validating the selective advantage granted by the HGT-mediated gain of the *fnr* gene by the strain A, which was one of the putative adaptations they identified through bioinformatics means earlier in the paper. Importantly, these experimental assays lend confidence to their earlier sequencing-based conclusions. They also describe an interesting vignette about how co-colonization can facilitate evolutionary modes that would potentially be impossible during a mono-colonization scenario, such as the restoration of a previously lost genomic locus via HGT between co-colonizing strains.

Major Critiques

There are several points that should be amended in subsequent revisions.

First, there is concern that the author's conclusions about the maintenance or loss of neutral polymorphism in the strain B1 and strain A of *E. coli* may be confounded by HGT. For example, while sweeps may appear hard in populations of the strain A, it is hypothetically possible that the locus bearing the fluorescent marker itself was transferred, while neutral variation is maintained elsewhere in the genome. Similarly, HGT may facilitate gene-specific sweeps of genetic variation in the strain B1 populations, even as the two distinct fluorescent markers are tagged. Put simply, the prevalence of HGT in your dataset raises the possibility that the fluorescent markers may do a poor job of tracking the fate of genetic variation (neutral or adaptive) at the genome-wide scale. This is a possibility that the authors are aware of and perhaps controlling for, as they write in the caption of Figure S2 that "HGT events must be invoked to explain marker fixation [in mice 7 and 8]." While the reviewers guess that the authors are using their pool sequencing results to rule out instances in which marker dynamics frequencies and genome-wide allele frequencies are at odds, it would be good to be explicit about how they made these judgements, as they seemed to do in the case of

mice 7 and 8. If they wish to only explain dynamics of the neutral marker, without making statements about whether neutral polymorphisms genome-wide are maintained or lost, then the authors should be more clear about this.

Second, the paper falls short of demonstrating evolutionary dynamics that are unique to scenarios in which conspecific strains co-colonize the same host. While it is clear how co-colonization between phylogenetically distinct strains might facilitate evolutionary events such as horizontal gene transfer (HGT), I think this study makes too strong of a claim about coexistence being a causative mechanism for much of the other evolutionary events they observe. For example, in the conclusion, they write "The co-existence can lead to intra-strain convergent evolution" (lines 309-310). This line explicitly states a more implicit framing of the entire narrative: that the dynamics observed herein are due to co-colonization. However, the authors do not adequately show that the evolutionary dynamics observed here are specific to a co-colonization scenario, as opposed to some more mundane explanation (e.g., differences in population size between the two strains, which may be relevant in a mono-colonization scenario as well). To the contrary, they state that many of the targets of adaptation identified in this study were also found in previous ones, "suggesting that these targets are independent of the co-colonization of strain A and B1 " (lines 161-162). If they had wanted to make a claim that tempo and mode of evolution is different during co-colonization compared to mono-colonization, they would have needed to explicitly compare their results to those observed in a mono-colonization scenario. This doesn't necessarily have to involve running more experiments, as the Gordo lab has a wealth of previous studies to draw from in trying to differentiate the dynamics observed here from those observed during a mono-colonization scenario (e.g., Barroso-Batista et al., 2014; Frazão et al., 2022; Frazão et al., 2023; etc.).

Third, it was a bit confusing how the simulation analysis advanced the study's central narrative. In general, the hypothesis and model being tested were not clear. For example, the abstract and conclusion state that there are "strain-specific selection dynamics," while the simulations that are apparently consistent with the data have all yield population scale mutation rates $\theta \sim 1 - 100$, which are consistent with very soft sweeps (for both populations). Therefore, it is unclear why this simulation should adequately describe dynamics observed in both strains, even though strain A is ostensibly governed by a regime of hard sweeps. Furthermore, some of the conclusions put forth run counter to empirical findings, even though the authors say they do. For instance, they state that they "would expect a higher rate of evolution in the strain B1... lead[ing] to a faster rate of loss of the neutral marker in the strain B1. These expectations are corroborated by simulations" (lines 105-108). However, strain B1 *did not* lose its neutral marker more quickly (Figure 1). In addition, certain assumption could have been better motivated, such as the use of a population size $N = 10^6$, which

seems small for bacterial populations in the gut, or the use of a gamma distribution to approximate the DFEs (while the reviewers are aware that this distribution is commonly used for this purpose, many readers may not). Finally, the simulations do not consider HGT, despite subsequent analyses showing this to be an important diversifying (and diversity-maintaining) evolutionary force. Overall, the thought is that the simulation could have been better motivated and explained, or left out of the main text altogether.

Finally, the reviewers suggest that the authors make it clear how this work is different from Frazão et al., 2022, as that study—which is more comprehensive—includes some similar findings (e.g., an HGT event between an endogenous strain of *E. coli* and an invader strain of *E. coli*).

Minor Critiques/confusions

- Hard versus soft sweep language:
    - Be careful with the usage of "hard sweep" and "soft sweep." In some contexts, the use of "complete sweep" and "partial sweep" seem to be more appropriate. Below are the definitions of all four types of sweeps as the reviewers understand them.
        - Hard sweep: a mutation with a single origin sweeping to high frequency.
        - Soft sweep: a mutation with multiple origins sweeping to high frequency, thereby increasing the frequency of more than one haplotype which harbor the origins.
        - Complete sweep: A mutation going to 100% frequency. This can include both hard and soft sweeps, depending on the number of origins giving rise to these mutations.
        - Partial sweep: a mutation going to intermediate frequency. Again, this term is agnostic to the number of origins.
    - For example, the authors label sweeps "soft" on several occasions (e.g., line 164), but because they have not shown the existence of multiple origins in the same host, "partial sweep" would be a more appropriate label.
- Authors should clearly define what they mean by *mutational parallelism* and *convergent evolution*. For example, it seems as if mutations arising in different strains in the same operon (as opposed to at the same site, or even within the same gene) is considered convergent evolution. Moreover, describing how "mutation parallelism at corresponding day" and "gene parallelism at corresponding day" included in table S2 is computed should be elaborated on. The reviewers could not find a definition for these terms in methods either.

- More could be said about the biological importance of Rac and Rac-like prophages (e.g., in the introduction)
- Early mention of phage piracy in the abstract with no introduction of it. It might be nice to explain to the reader what this is.
- The authors should say why neutral polymorphisms are important somewhere in the introduction or results, as it may not be immediately clear to a reader
- How was read contamination detected?
- Do instances of convergence between the two phylogenetically distinct strains occur in the same mouse?
    - It seems like they do for some genes (i.e., those in the fim operon). I think this should be mentioned in the main text, as it has interesting implications (e.g., for whether we expect strains to undergo similar evolutionary trajectories if they're in the same host versus different hosts).
- How did the authors identify *fnr* as an important gene that necessitated followup experiments? Were other genes that were gained during the transfer event from B1 to A not core genes? If no other gene that was gained during the HGT event was core, it would have helped explain your reasoning for performing followup functional validation on the *fnr* locus
- When was sequencing performed on a single clone, and why (line 682)? This was unclear to the reviewers, even though it may have a significant impact on the interpretation of the results.
- Does strain A vs Strain B have the smaller genome? Could that explain the larger number of mutations? Is M(t) normalized by the number of basepairs?
- Figures suggestions
    - Figure 1:
        - Y axis label is not formatted correctly
        - Legend should make it clear which colors are associated with strains A and B1
        - This suggestions also holds for figure S2—each strain should be connected to their associated fluorescent markers in a legend
    - Figure 2:
        - 2A: Where is fadL (which supposedly shows convergent evolution) in strain A? Is evolution in fadL and fadH considered convergent evolution? It seems like not all targets of evolution are shown in the plot. Why not?
    - Figure 3:
        - Y-axis of A/B and C/D do not have the same ranges. It would be nice if they had comparable ranges to make the differences in carrying capacity and growth rate more visually comparable.
    - Fig 4C:

        - why is it important to test in 2 male and 2 female mice? However, there does seem to be some sex interaction – how does this affect the rest of the results with the other 8 mice? Are all male? All female?
- Typos/formatting errors:
    - line 93-95
    - line 288
