# Supplementary material for: Clonal interference and genomic repair during strain coexistence in the gut
Source: PLoS Genet. 2025 Jul 7;21(7):e1011777. doi: 10.1371/journal.pgen.1011777 (PMC12251178; doi:10.1371/journal.pgen.1011777)
Supplement: Attachment — Submitted filename: Response_to_Reviewers.docx [file pgen.1011777.s023.docx]

***Dear Editors,***

***We thank the reviewers for their important comments, which we address point by point below highlighted in blue and with the indication of the corresponding lines in the Revised Manuscript.***

**Reviewers' comments:**

**Reviewer #1:** Clonal Interference and genomic repair

during strain coexistence in the gut

Frazão et al., PLOS Genetics

Summary

This paper sets out to investigate the mode and tempo of evolution displayed by two co-colonizing, phylogenetically distinct strains of Escherichia coli in the mouse gut.

Little is known about how evolution proceeds when two conspecific stains of the same bacterial species coexist in the same host gut. Frazão et al. find that the two strain populations can be distinguished according to their evolutionary dynamics: a mouse-associated strain (strain B1), which colonizes the mice at a significantly greater population size than the laboratory strain (strain A), shows a pattern consistent with clonal interference, whereby adaptive mutations sweep to intermediate frequency via partial sweeps (not necessarily “soft sweeps,” as they are called in the paper, as elaborated on further below) and neutral variation was maintained. Contrastingly, evolution in the populations of a laboratory strain that colonized the mice is characterized by hard, complete sweeps in which adaptive mutations sweep to fixation, thereby eliminating neutral variation from the population. The study also finds evidence for extensive horizontal gene transfer (HGT) in which genetic material is transferred from the mouse-associated strain to the laboratory strain. Strikingly, one of these transfers restored genes important for gut colonization to the laboratory strain which that strain had previously lost during in vitro passaging. Finally, the study identifies what is likely the first documented instance of an in vivo transfer of a phage satellite—a mobile genetic element that exploits machinery of other prophages to transfer between cells.

Positives

This study addresses a significant gap in the literature by examining how bacterial strains evolve when co-colonizing the gut of the same host, as most previous research focuses on the evolutionary trajectories displayed by single strains. In pairing a fluorescence-based approach with pool sequencing, they are able to describe broadscale patterns in the evolutionary regimes operating on the two distinct strains (i.e., an evolutionary regime characterized by complete versus partial sweeps, respectively) as well as the specific loci that are under selection and responsible for producing these broad-scale patterns. Altogether, this narrative they present is interesting and thoroughly told. Moreover, the authors are able to offer convincing evidence that the evolutionary changes they document are likely adaptive, including by detecting widespread instances of parallelism between technical replicates of the same strain and evolutionary convergence between strains. It is quite an interesting finding that although the distinct strain populations show overlapping targets of adaptation, the manner in which they evolve is strain-specific—characterised by either partial or hard/complete sweeps, depending on the strain. Finally, the authors do their due diligence in functionally validating the selective advantage granted by the HGT-mediated gain of the fnr gene by the strain A, which was one of the putative adaptations they identified through bioinformatics means earlier in the paper. Importantly, these experimental assays lend confidence to their earlier sequencing-based conclusions. They also describe an interesting vignette about how co-colonization can facilitate evolutionary modes that would potentially be impossible during a mono-colonization scenario, such as the restoration of a previously lost genomic locus via HGT between co-colonizing strains.

*We thank the reviewer for the summary of the paper and the positive comments.*

Major Critiques

There are several points that should be amended in subsequent revisions.

First, there is concern that the author’s conclusions about the maintenance or loss of neutral polymorphism in the strain B1 and strain A of E. coli may be confounded by HGT. For example, while sweeps may appear hard in populations of the strain A, it is hypothetically possible that the locus bearing the fluorescent marker itself was transferred, while neutral variation is maintained elsewhere in the genome.

Similarly, HGT may facilitate gene-specific sweeps of genetic variation in the strain B1 populations, even as the two distinct fluorescent markers are tagged. Put simply, the prevalence of HGT in your dataset raises the possibility that the fluorescent markers may do a poor job of tracking the fate of genetic variation (neutral or adaptive) at the genome-wide scale. This is a possibility that the authors are aware of and perhaps controlling for, as they write in the caption of Figure S2 that “HGT events must be invoked to explain marker fixation [in mice 7 and 8].” While the reviewers guess that the authors are using their pool sequencing results to rule out instances in which marker dynamics frequencies and genome-wide allele frequencies are at odds, it would be good to be explicit about how they made these judgements, as they seemed to do in the case of mice 7 and 8. If they wish to only explain dynamics of the neutral marker, without making statements about whether neutral polymorphisms genome-wide are maintained or lost, then the authors should be more clear about this.

*We thank the reviewer for this important comment. We would like to note that each fluorescent marker is located near a distinct antibiotic resistance gene; therefore, if fluorescence transfer had occurred we would also expect a transfer of the corresponding antibiotic resistance gene, a phenomenon that we did not observe. Furthermore, each fluorescently tagged strain carries unique strain-specific mutations, and the frequencies of these diagnostic mutations are consistent with those of their respective markers. We have now clarified this point in the Methods section (lines 376-380):* *“To exclude that transfer of fluorescent markers between strains occurred, we examined the frequencies of strain-specific mutations unique to each fluorescent strain (S12 Table). The frequency of these mutations was consistent with their respective markers, suggesting that transfer of fluorescent markers between strains did not occur.”.*

*To ensure clarity regarding our definition of neutral polymorphism, we have revised the abstract (lines 18-21) to state that polymorphism was measured at a neutral locus: “We find qualitative differences in evolutionary dynamics between strains within the same host. In the strain with larger population size intense clonal interference occurs and polymorphism at a neutral marker locus is maintained, while in the strain with lower population size complete selective sweeps and loss of neutral marker polymorphism occurs.”. Additionally, we have also modified the caption of S2 Fig to read: “… HGT events in other genomic regions must be invoked to explain neutral fluorescent marker fixation (S2 Table).”.*

Second, the paper falls short of demonstrating evolutionary dynamics that are unique to scenarios in which conspecific strains co-colonize the same host. While it is clear how co-colonization between phylogenetically distinct strains might facilitate evolutionary events such as horizontal gene transfer (HGT), I think this study makes too strong of a claim about coexistence being a causative mechanism for much of the other evolutionary events they observe. For example, in the conclusion, they write “The co-existence can lead to intra-strain convergent evolution” (lines 309-310). This line explicitly states a more implicit framing of the entire narrative: that the dynamics observed herein are due to co-colonization. However, the authors do not adequately show that the evolutionary dynamics observed here are specific to a co-colonization scenario, as opposed to some more mundane explanation (e.g., differences in population size between the two strains, which may be relevant in a mono-colonization scenario as well). To the contrary, they state that many of the targets of adaptation identified in this study were also found in previous ones, “suggesting that these targets are independent of the co-colonization of strain A and B1 ” (lines 161-162). If they had wanted to make a claim that tempo and mode of evolution is different during co-colonization compared to mono-colonization, they would have needed to explicitly compare their results to those observed in a mono-colonization scenario. This doesn’t necessarily have to involve running more experiments, as the Gordo lab has a wealth of previous studies to draw from in trying to differentiate the dynamics observed here from those observed during a mono-colonization scenario (e.g., Barroso-Batista et al., 2014; Frazão et al., 2022; Frazão et al., 2023; etc.).

*We thank the reviewer for this valuable comment and fully agree with their point. The sentence that implied coexistence as the cause of the observed evolutionary events, which we do not want to claim, has now been rewritten in the Conclusion section (lines 350-351) as follows: “Intra-strain convergent evolution was observed and large amounts of genetic information were exchanged from one strain to the other, …”.*

Third, it was a bit confusing how the simulation analysis advanced the study’s central narrative. In general, the hypothesis and model being tested were not clear. For example, the abstract and conclusion state that there are “strain-specific selection dynamics,” while the simulations that are apparently consistent with the data have all yield population scale mutation rates θ ~ 1 − 100, which are consistent with very soft sweeps (for both populations). Therefore, it is unclear why this simulation should adequately describe dynamics observed in both strains, even though strain A is ostensibly governed by a regime of hard sweeps. Furthermore, some of the conclusions put forth run counter to empirical findings, even though the authors say they do. For instance, they state that they “would expect a higher rate of evolution in the strain B1… lead[ing] to a faster rate of loss of the neutral marker in the strain B1. These expectations are corroborated by simulations” (lines 105-108). However, strain B1 did not lose its neutral marker more quickly (Figure 1). In addition, certain assumption could have been better motivated, such as the use of a population size 𝑁 = 10 , which seems small for bacterial populations in the gut, or the use of a gamma distribution to approximate the DFEs (while the reviewers are aware that this distribution is commonly used for this purpose, many readers may not). Finally, the simulations do not consider HGT, despite subsequent analyses showing this to be an important diversifying (and diversity-maintaining) evolutionary force. Overall, the thought is that the simulation could have been better motivated and explained, or left out of the main text altogether.

*We thank the reviewer for this comment. To clarify our motivation and better explain our simulation analysis, we have now revised the section regarding the simulations (lines 130-144) which know reads as follows: “Given the large differences in the density of each strain and previous theoretical work suggesting that the probability of fixation of beneficial mutations in clonal populations depends on population size [22], we decided to explore conditions under which a model of positive directional selection in strictly clonal populations could explain the experimental data on strain coexistence and the maintenance or loss of polymorphism at a neutral locus. We performed simulations assuming a simple evolutionary model where adaptive mutations are unconditionally beneficial; the distribution of their effects (DFEBM) is similar between the strains and no gene transfer from one strain to another can occur. Simulations across a wide range of values for the mutation rate show that when the DFEBM is exponential (a commonly used assumption [23]), the model does not explain the experimentally observed data (see S3 Fig). However, the observed maintenance vs loss of neutral marker polymorphism in each strain could be seen under other DFEBMs, but only under particular conditions. Specifically, the simulations show that a model of positive directional selection is only compatible with the observed data (Fig 1B) if all beneficial mutations have similar effects (~1%) (S3 Fig, left panel).”*

Finally, the reviewers suggest that the authors make it clear how this work is different from Frazão et al., 2022, as that study—which is more comprehensive—includes some similar findings (e.g., an HGT event between an endogenous strain of E. coli and an invader strain of E. coli).

*We thank the reviewer for this helpful suggestion. To better distinguish this work from that of Frazão et al., 2022, we have now clarified the distinction in the Introduction section (lines 55-67) where we write: “In our previous work we found that when an invader E. coli strain was introduced in the gut environment of mice it was able to coexist with a E. coli resident already present in their microbiota. Furthermore, events of horizontal gene transfer (HGT) from the resident strain to the invader strain, driven by either bacteriophages or conjugative plasmids [12], could be detected. We also found that the acquisition of temperate bacteriophages, such as Rac-like prophages, by the invader strain conferred a metabolic advantage to the bacterial host [13,14]. However, the extent to which rates of adaptation vary across E. coli strains coexisting in the same host is still poorly known, as evolutionary dynamics are typically followed in a single colonizer strain. This was also the case in our previous work where one the E. coli strains was already present in the mouse gut [12,14]. Here, we take an in vivo experimental evolution approach of controlled co-colonization, using those two E. coli strains, enabling a direct comparison of each strain evolutionary dynamics.”.*

Minor Critiques/confusions

● Hard versus soft sweep language:

○ Be careful with the usage of “hard sweep” and “soft sweep.” In some contexts, the use of “complete sweep” and “partial sweep” seem to be more appropriate. Below are the definitions of all four types of sweeps as the reviewers understand them.

■ Hard sweep: a mutation with a single origin sweeping to high frequency.

■ Soft sweep: a mutation with multiple origins sweeping to high frequency, thereby increasing the frequency of more than one haplotype which harbor the origins.

■ Complete sweep: A mutation going to 100% frequency. This can include both hard and soft sweeps, depending on the number of origins giving rise to these mutations.

■ Partial sweep: a mutation going to intermediate frequency. Again, this term is agnostic to the number of origins.

○ For example, the authors label sweeps “soft” on several occasions (e.g., line 164), but because they have not shown the existence of multiple origins in the same host, “partial sweep” would be a more appropriate label.

*We thank the reviewer for the suggestion, which we agree. We have now replaced throughout the manuscript “hard sweep” by “complete sweep” (lines 21, 100, 171, and 201), and “soft sweep” by “partial sweep” (lines 106, 195, and 198).*

● Authors should clearly define what they mean by mutational parallelism and convergent evolution. For example, it seems as if mutations arising in different strains in the same operon (as opposed to at the same site, or even within the same gene) is considered convergent evolution. Moreover, describing how “mutation parallelism at corresponding day” and “gene parallelism at corresponding day” included in table S2 is computed should be elaborated on The reviewers could not find a definition for these terms in methods either.

*We have now introduced in the manuscript our definitions of mutational parallelism and convergent evolution. In lines 158-160 it now reads: “Twelve targets of parallel evolution (i.e. mutational events in the same target observed in different mice) were identified in strain B1 (Fig 2B and S3 Table), two of which correspond to large deletions.”; and in lines 220-222 it now reads: “In the two phylogenetic distinct strains studied we found that several targets shared between strains underwent convergent evolution (i.e. mutational events in the same gene or pathway observed in both strain A and strain B1).”. We have also modified the legends of S2 and S3 Tables to include our definition of “mutation parallelism at corresponding day” and “gene parallelism at corresponding day”. It now reads: “Mutation parallelism at corresponding day indicates the number of mice in which the exact same mutation was detected at day 43 or day 88. Gene parallelism at corresponding day indicates the number of mice in which mutational events targeting the same gene were detected at day 43 or day 88.”.*

● More could be said about the biological importance of Rac and Rac-like prophages (e.g., in the introduction)

*We have now introduced a sentence in the Introduction section where we mention the biological importance Rac-like prophages. In lines 59-61 it now reads: “We also found that the acquisition of temperate bacteriophages, such as Rac-like prophages, by the invader strain conferred a metabolic advantage to the bacterial host [13,14].”*

● Early mention of phage piracy in the abstract with no introduction of it. It might be nice to explain to the reader what this is.

*We have now modified the abstract by introducing the definition of phage piracy. In lines 24-26 it now reads: “Furthermore, we detect for the first time a case of phage piracy in the gut, where a putative phage satellite, lacking essential genes for their own replication, was likely mobilized by a helper phage to transfer between bacterial hosts.”*

● The authors should say why neutral polymorphisms are important somewhere in the introduction or results, as it may not be immediately clear to a reader

*We have now modified the section “Coexistence of E. coli strains in the mouse gut and strain dependent mode of evolution” to highlight the importance of neutral fluorescent marker polymorphisms.*

● How was read contamination detected?

*We have now clarified in the Methods section “Bioinformatic analysis of sequenced evolved populations, evolved and ancestral clones” the strategy used for detecting read contamination. In lines 461-470 it now reads: “Variant calling on the samples from mouse 8 (day 43 and 88) for strain A and strain B1 revealed thousands of variants across the reference genome. Using BLASTN [54], we identified reads with 100% identity with genomes from Enterobacter hormachei. For these samples, BBSplit was run including genomes from E. hormachei (Accession Numbers: CP104691.1, CP104689.1, CP056649.1, CP044335.1, CP051132.1, CP116960.1, and CP019889.1). The seven genomes for E. hormachei were selected based on a 100% match of the contaminant reads with NCBI database. A similar pattern of thousands of variants across the reference genome of strain B1 was also observed in the samples from mouse 4 (day 43 and 88) and from mouse 6 (day 43). However, in this case BLASTN analysis revealed a 100% identity with the genome of strain A.”*

● Do instances of convergence between the two phylogenetically distinct strains occur in the same mouse? It seems like they do for some genes (i.e., those in the fim operon). I think this should be mentioned in the main text, as it has interesting implications (e.g., for whether we expect strains to undergo similar evolutionary trajectories if they’re in the same host versus different hosts).

*We have now commented on this on line 225-231 as follows: “Consistent with an environment where different E. coli strains likely compete for niches and/or resources, the evolutionary convergent targets are related to motility/adhesion through type 1 fimbriae (fimA, fimE, fimH, fimI) [28], with these fim targets found in E. coli populations colonizing the same host (mice 1 and 7). Convergent targets were also observed in central carbon metabolism (barA and uvrY) [29], fatty acid degradation (fadH and fadL) in mouse 2 [30], and threonine/serine metabolism (tdcA) [31].”*

● How did the authors identify fnr as an important gene that necessitated followup experiments?

*We have now clarified in the Methods section the reasoning for performing follow up experiments with fnr. In lines 489-495, it now reads: “Remarkably, the evolved populations from mouse 7 displayed a distinct pattern: numerous potential HGT variants extended into the non-prophage region flanking the Rac prophage were detected in this mouse (S7 Table). This suggests that a substantial genomic region was horizontally transferred from strain B1 to strain A. Importantly, this region included the fnr gene, essential for anaerobic respiration and known to contribute to increased fitness in the gut, which is absent in strain A but was restored in the evolved population of strain A from mouse 7.”.*

Were other genes that were gained during the transfer event from B1 to A not core genes? If no other gene that was gained during the HGT event was core, it would have helped explain your reasoning for performing followup functional validation on the fnr locus.

*We have now clarified the sentence mentioned by the reviewer. In lines 300-307, it now reads: “The functions repaired in strain A include: motility, adhesion, and biofilms (uspE [36] and ydaM [37]); stress response and DNA repair (uspE [36] and ogt [38]); metabolite transport (abg operon [39] and ydaN [40]); and the switch from aerobic to anaerobic metabolism (fnr [41]). The latter is particularly interesting as fnr is an important gene for the ability of E. coli to colonize the mammalian gut. In fact, the presence of fnr in the genome of this bacterium is known to provide a fitness advantage in the mouse gut, as demonstrated by competitive fitness assays between fnr KO mutants and wild-type clones across different genomic backgrounds of E. coli [42].”.*

● When was sequencing performed on a single clone, and why (line 682)? This was unclear to the reviewers, even though it may have a significant impact on the interpretation of the results.

*We have now clarified in the Methods when sequencing was performed on single clone. In lines 422-424 it now reads: “DNA extraction for Illumina sequencing. DNA was extracted with Phenol-Chloroform [49] from E. coli populations (mixture of >1000 clones) from day 43 and day 88 after growing in LB plates supplemented with antibiotic to avoid contamination.”; and in lines 590-592 it now reads: “Nanopore sequencing of fnr-positive clones and analysis. DNA was extracted as described above from two fnr-positive clones of strain A (confirmation by PCR, see above) isolated from mouse 7 at day 88 post-colonization.”. Furthermore, we modified lines 281-286 in the Results section “Horizontal Gene Transfer between strains of E. coli repairs an important K12 gene” to further clarify the point raised by the reviewer, which now reads: “Illumina sequencing of the E. coli populations of strain A and strain B1 at days 43 and 88 suggested a remarkable case of hybridization between coexisting strains in one of the mice (mouse 7). To validate this finding, we performed Nanopore long-read sequencing on two clones of strain A isolated from mouse 7 at day 88 (S6, S7 and S8 Tables), which confirmed the acquisition of a large genomic region in the chromosome of strain A. Specifically, a 93,712 bp region in strain A was replaced by a 120,546 bp region from strain B1 (Fig 4B).”; and the legend for S8 Table, which now reads: “S8 Table. List of mutations obtained from the Breseq output for 2 fnr-positive clones from strain A from mouse 7 at day 88 after Nanopore sequencing.”.*

● Does strain A vs Strain B have the smaller genome? Could that explain the larger number of mutations? Is M(t) normalized by the number of basepairs?

*Strain A has a smaller genome. We now have added on line 93-95 the following sentences: “The latter belongs to phylogenetic group A, is the best characterized E. coli strain and a good recipient of MGEs from the donor strain. Its genome is smaller than that of mouse resident strain. We will refer to the mouse commensal strain as “strain B1” and to K12 as “strain A”.”.*

*M(t) is not normalized by the number of basepairs. We now clarify on lines 240-243, which now reads: “Following Good et al. [33], we estimated the genomic rate of mutation accumulation (M(t)) by summing the allele frequencies of all the mutations detected in each strain, after three months of co-colonization, and dividing this value by the number of elapsed generations (1584 generations, assuming 18 generations per day similar for each strain).“*.

● Figures suggestions

○ Figure 1:

■ Y axis label is not formatted correctly

*We have now formatted correctly the Y axis label of Figure 1B.*

■ Legend should make it clear which colors are associated with strains A and B1

*We have now modified Fig 1B to indicate which panels correspond to strain A and strain B1, and clarified in the legend to which strains each color is associated. In lines 831-834 it now reads: “In each cohort, mice were co-colonized with two fluorescent variants of strain A (CFP, blue circles; YFP, yellow circles) and two fluorescent variants of strain B1 (mCherry, red circles; GFP, green circles) E. coli, to follow their evolution in the mouse gut (see S1 Fig for more details).”.*

■ This suggestions also holds for figure S2—each strain should be connected to their associated fluorescent markers in a legend

*We have now modified S2 Fig and its corresponding legend as described above for Fig 1B.*

○ Figure 2:

■ 2A: Where is fadL (which supposedly shows convergent evolution) in strain A? Is evolution in fadL and fadH considered convergent evolution? It seems like not all targets of evolution are shown in the plot. Why not?

*We have now clarified in the manuscript how we define convergent evolution, in lines 220-222 it now reads: “In the two phylogenetic distinct strains studied we found that several targets shared between strains underwent convergent evolution (i.e. mutational events in the same gene or pathway observed in both strain A and strain B1).”. Using this definition, mutations in fadL and fadH are considered convergent evolution as they share the same pathway – fatty acid degradation. Figure 2A and 2B show all the targets of evolution observed at day 88. We have now modified the Figure 2 legend for clarification, in lines 840-841 it now reads “Circular plots indicating the targets of evolution along the genome of strain A (A) and strain B1 (B) obtained from population sequencing at day 88.”.*

○ Figure 3:

■ Y-axis of A/B and C/D do not have the same ranges. It would be nice if they had comparable ranges to make the differences in carrying capacity and growth rate more visually comparable.

*We have now modified the Y-axis of Figure 3 to show the same range.*

○ Fig 4C:

■ why is it important to test in 2 male and 2 female mice? To generalize the results. However, there does seem to be some sex interaction – how does this affect the rest of the results with the other 8 mice? If true it means that in males the fnr provides a bigger advantage than in females. Are all male? All female?

*We did not design the experiment of the competitive fitness assay with sufficient power to address if there were significant fitness differences between males and females. Our intention was just to test if the advantage existed in females (the sex of the evolution experiment) and if there was also an advantage in males. We note that the co-housing during the one-week treatment with streptomycin prior to the colonization was possible in the two males used because they were from the same litter (If they were not siblings, aggressive interactions would likely have occurred during co-housing). We have now clarified in the methods that all mice in the evolution experiment were female (line 384); and in the beginning of the section “Coexistence of E. coli strains in the mouse gut and strain dependent mode of evolution”, line 89. We also re-wrote the section “ Competitive fitness assay in vivo.”, lines 540-544, as follows “The relative fitness of an evolved E. coli clone of strain A, bearing the Nef and KingRac prophages, as well as the horizontally transferred fnr gene region (clone 7, S9 Table), was measured in the guts of mice (n=4). Although the evolution experiment used only female mice, the competitive fitness assay included two female and two male mice to test for a sex independent fitness advantage.”.*

● Typos/formatting errors:

○ line 93-95

*We have now corrected the typo. In lines 117-120 it now reads: “Temporal series data of the abundance of each E. coli strain in the co-colonized mice shows that both strains can coexist for at least three months in the mouse gut (coexistence observed in eight out of nine mice, strain A failed to colonize in mouse 9, Fig 1B, S1 Fig, and S1 Table).”.*

○ line 288

*We have now corrected the typo. In lines 328-330 it now reads: “Specifically the gene ash, which has a homologue in the P4 satellite and is a core gene of the P4-like family of satellites [43].”.*

**Reviewer #2:** Understanding how different strains compete and coexist within a specific niche, as well as uncovering the molecular mechanisms driving such competition, represents a fascinating area of research. In the current manuscript, the authors address these compelling questions by analysing the competition and evolution of two E. coli strains in the gut environment. Overall, I find the manuscript highly engaging, particularly the sections where the authors describe several clear examples of horizontal gene transfer (HGT), including the transfer of large portions of the bacterial chromosome and a putative phage satellite. These findings provide valuable insights into microbial evolution and inter-strain interactions.

*We thank the reviewer for the positive comments.*

While I have no major criticisms, I offer the following suggestions to further improve the manuscript:

- Reorganisation of results. The initial part of the results, which outlines all the putative scenarios that might occur, detracts somewhat from the narrative. I suggest restructuring this section to first describe what actually occurs, followed by the corresponding explanations. The other hypothetical scenarios could be moved to the discussion section, where they might be better contextualised and explored.

*We have now re-organized the first section of the Results and the Introduction. Specifically, lines 83-91 from the original manuscript were rewrote and moved to the Introduction section corresponding now to lines 55-61: “In our previous work we found that when an invader E. coli strain was introduced in the gut environment of mice it was able to coexist with a E. coli resident already present in their microbiota. Furthermore, events of horizontal gene transfer (HGT) from the resident strain to the invader strain, driven by either bacteriophages or conjugative plasmids [12], could be detected. We also found that the acquisition of temperate bacteriophages, such as Rac-like prophages, by the invader strain conferred a metabolic advantage to the bacterial host [13,14].”.*

- In the same initial section, I found that some of the terminology, such as "neutral polymorphism," may be challenging for non-experts to grasp. Providing a clearer explanation of these terms would enhance the readability and accessibility of the manuscript for a broader audience.

*We thank the reviewer for this comment, also shared by reviewer #1. We have now modified the Results section to clarify the meaning of “neutral polymorphism”. Throughout this section, we have now replaced “neutral polymorphism” by “neutral fluorescent marker polymorphism”.*

- If I understand correctly, the authors performed pooled sequencing of the strains at the end of the experiments. However, I missed seeing individual sequencing data for specific clones. It remains unclear whether all clones from the same strain exhibit identical mutations or whether different clones harbour distinct mutations. Including a more detailed analysis or discussion of clone-specific genetic variation would greatly enrich the understanding of strain dynamics.

*We thank the reviewer for this comment. We have now clarified in the Methods when sequencing was performed on single clone. In lines 422-424 it now reads: “DNA extraction for Illumina sequencing. DNA was extracted with Phenol-Chloroform [49] from E. coli populations (mixture of >1000 clones) from day 43 and day 88 after growing in LB plates supplemented with antibiotic to avoid contamination.”; and in lines 590-592 it now reads: “Nanopore sequencing of fnr-positive clones and analysis. DNA was extracted as described above from two fnr-positive clones of strain A (confirmation by PCR, see above) isolated from mouse 7 at day 88 post-colonization.”. Furthermore, we modified lines 281-286 in the Results section “Horizontal Gene Transfer between strains of E. coli repairs an important K12 gene” to further clarify when sequencing of individual clones was performed, which now reads: “Illumina sequencing of the E. coli populations of strain A and strain B1 at days 43 and 88 suggested a remarkable case of hybridization between coexisting strains in one of the mice (mouse 7). To validate this finding, we performed Nanopore long-read sequencing on two clones of strain A isolated from mouse 7 at day 88 (S6, S7 and S8 Tables), which confirmed the acquisition of a large genomic region in the chromosome of strain A. Specifically, a 93,712 bp region in strain A was replaced by a 120,546 bp region from strain B1 (Fig 4B).”.*

- The manuscript mentions that mice were treated with antibiotics to establish colonisation. However, it is unclear how significantly this treatment reduces the microbiota and how natural the tested conditions are as a result. Additionally, it would be helpful to clarify whether other E. coli strains were present in the gut during the experiments and how they might influence the dynamics between the two focal strains.

*We thank the reviewer for the comment. We have now clarified in the manuscript and in the Methods section that we used a classical model for E. coli colonization that maintains a complex microbiota composition while eliminating resident E. coli. In lines 86-89 it now reads: “We used a classical model for E. coli colonization of the mouse gut, the streptomycin treated mouse model [16]. This treatment allows for the successful colonization by E. coli of the mouse gut while maintaining a complex microbiota composition [12], although with a reduced species diversity [17]. Briefly, female mice were simultaneously colonized with two E. coli strains…” and in lines 382-388 it now reads: “We used a classical mouse gut colonization model where C57BL/6J mice (Mus musculus) were supplied by the Rodent Facility at Gulbenkian Institute. Animals were kept co-housed to homogenize the mouse microbiota during treatment with streptomycin (5 g/L) in the drinking water for seven days and the following two days, streptomycin treatment was absent to clean the gut from antibiotic traces [48]. This classical colonization model allows successful colonization with E. coli, maintaining a complex microbiota composition and eliminating the resident E. coli.”.*

- While I recognise it may be too late to conduct additional experiments, it would be valuable for the authors to discuss the potential outcomes of growing the two strains together in a controlled in vitro setting, such as in a nutrient-rich medium. Comparing the trajectories of strain competition and evolution under such conditions could provide further insights into the role of the gut environment in shaping the observed dynamics.

*We thank the reviewer for this comment. Although out of the scope of the current manuscript, we agree that it would be interesting to compare the trajectories of both strains in controlled in vitro settings. However, given the large number of in vitro settings that can be used to address this question, it is our opinion that characterizing these dynamics in in vivo conditions can contribute for defining the ideal in vitro settings.*

These suggestions aim to enhance the clarity, comprehensiveness, and impact of the manuscript. The study presents intriguing findings, and addressing these points will further strengthen its contribution to the field.

**Reviewer #3:** This manuscript presents the results of a co-colonization experiment of two E. coli strains in mice. The authors use fluorescent markers as a proxy for neutral genetic diversity in the two strains, one which is native to the mouse gut (B1) and the other the laboratory K12 strain (A). They track the evolution of both strains across 9 mice over approximately three months.

The authors find repeated mutations across mice in both strains, suggesting they are both undergoing adaptive evolution. They also observe several HGT events from B1 to A. The gene functions in these segments and competitive fitness assays suggest these HGT events were beneficial in A and restored functions that were previously lost in A.

The authors also characterize the rate of adaptation in the two strains and observe some interesting differences. Somewhat surprisingly, they detect a larger average number of mutations in the native gut strain B1 compared to the laboratory strain A (~10 vs ~5), which they attribute to the higher population size of the B1 strain, as measured by CFU/ml. However, they do not detected any difference in the rate of mutation accumulation in the two strains. In addition, they observe the fixation of one of the A fluorescent markers in all mice that successfully colonized, but no fixation in B1.

The results of the manuscript are new to my knowledge. The observation of convergent evolution during co-colonization and the large impact of HGT in the initial adaptation process is especially noteworthy and could be relevant for many other bacteria, including in natural populations [1, 2]. The manuscript is well-written and the results and methods clearly presented.

[1] Sheppard, SK, et al., Convergence of Campylobacter Species: Implications for Bacterial Evolution, Science 320, 237-239 (2008).

[2] Birzu, G, et al., Hybridization breaks species barriers in long-term coevolution of a cyanobacterial population, bioRxiv:2023.06.06.543983 (2023)

*We thank the reviewer for the positive comments and the relevant note on the importance of our work for other bacteria, which we now mention in the Conclusion section. In lines 357-359 it now reads: “Furthermore, our observations on convergent evolution during co-colonization and the large impact of HGT could be relevant for other bacterial species in natural populations [46,47].”.*

My only major comment has to do with the explanation for the loss of diversity in the A strain. Specifically, I was not convinced by the explanation of the authors that the lower population size of A would lead to hard sweeps but not in B1. First, do the authors have estimates of the population sizes of the strains in the mouse gut? Naively, I would expect the both strains to be in a high mutation supply regime (N\mu > 1). Also, even if the B1 population has more clonal interference, the fluorescent label should still be lost eventually, in which case there may be a quantitative but not qualitative difference between the two strains. It was also not clear to me how the rate of mutation accumulation in the two strains is similar but the average number of mutations in the strains differ by around a factor of 2.

*We thank the reviewer for the comment. We have now modified the abstract according to the reviewer suggestion. In lines 18-21 it now reads: “We find qualitative differences in evolutionary dynamics between strains within the same host. In the strain with larger population size intense clonal interference occurs and polymorphism at a neutral marker locus is maintained, while in the strain with lower population size complete selective sweeps and loss of neutral marker polymorphism occurs.”. Furthermore, in the section “Genomic landscape of adaptive evolution of coexisting E. coli strains”, we have also re-written some sentences in the 3^rd^ paragraph, specifically on lines 195-201 we now write: “Interestingly, there were less detected partial sweeps in strain A, relative to strain B1, during the period in which evolution was followed. Multiple alleles segregating at the same locus were detected only in one out of eight mice for strain A vs. six out of eight for strain B1, after the 88 days of co-colonization. At an intermediate point (43 days of co-colonization) partial sweeps were detected for strain A in two out of eight mice vs. four out of eight mice for strain B1. This suggests strain A experienced less clonal interference than strain B1 throughout the entire evolution period.”. The rate of mutation accumulation M(t) was calculated by weighting the frequencies of each mutation (a sentence stating this is on lines 240-243), whereas the number of mutations does not take their frequency into account. As strain B1 evolutionary dynamics are driven by intense clonal interference and partial sweeps and strain A by complete sweeps, we observe a similar rate of mutation accumulation between both strains but a higher number of mutations in strain B1.*

Minor comments

- L. 114: I did not understand what is meant here by "much lower variance than an exponential." The variance of an exponential distribution is determined by its decay rate.

*We have now modified the paragraph in which the simulations are discussed for clarification. In lines 130-144, it now reads: “Given the large differences in the density of each strain and previous theoretical work suggesting that the probability of fixation of beneficial mutations in clonal populations depends on population size [22], we decided to explore conditions under which a model of positive directional selection in strictly clonal populations could explain the experimental data on strain coexistence and the maintenance or loss of polymorphism at a neutral locus. We performed simulations assuming a simple evolutionary model where adaptive mutations are unconditionally beneficial; the distribution of their effects (DFEBM) is similar between the strains and no gene transfer from one strain to another can occur. Simulations across a wide range of values for the mutation rate show that when the DFEBM is exponential (a commonly used assumption [23]), the model does not explain the experimentally observed data (see S3 Fig). However, the observed maintenance vs loss of neutral marker polymorphism in each strain could be seen under other DFEBMs, but only under particular conditions. Specifically, the simulations show that a model of positive directional selection is only compatible with the observed data (Fig 1B) if all beneficial mutations have similar effects (~1%) (S3 Fig, left panel).”.*

- L. 122: It would be useful if the authors could comment on the rate of increase in mutations compared to other measurements. For example, it seems like the numbers reported here are fairly similar to Lenski's LTEE.

*We have now commented on the similarity of the rates of evolution with those in Lenski experiment: On line 254-256 it now reads: “Interestingly, the rates of evolution estimated here are very similar to those observed in the initial period of evolution of the in vitro propagation of a E. coli strain in minimal media with glucose [33].”.*

- Fig. 1: The y-axis label is distorted.

*We have now corrected the distortion in the y-axis label of Fig 1B.*
