# Supplementary material for: Clonal interference and genomic repair during strain coexistence in the gut
Source: PLoS Genet. 2025 Jul 7;21(7):e1011777. doi: 10.1371/journal.pgen.1011777 (PMC12251178; doi:10.1371/journal.pgen.1011777)
Supplement: Attachment — Submitted filename: Response_to_Reviewers_auresp_2.docx [file pgen.1011777.s024.docx]

**Response to Reviewers**

**Reviewer #1**: We appreciate the author’s openness to our questions and critiques and their good-faith (and extensive) attempts to resolve them. We believe that the most recent revisions to the manuscript have significantly elevated the quality of the work.

*We thank Reviewer 1 for their valuable efforts in reviewing our manuscript.*

However, there are several points we urge the authors to address prior to publication:

1. While the authors have successfully clarified that the simulations are meant to rule population size as a potential mechanism explaining differences in the evolutionary dynamics observed in populations of strains A and B1, respectively, we still question whether the simulations successfully achieve this aim. If the simulations ultimately are included in the main text: **(1)** the authors should explain why they heed simulations using models with exponentially distributed effects more than models which assign a uniform distribution of effects (S ~ 1%) to mutations, particularly as population size was able to explain differential dynamics observed in the empirical data under these latter models; and **(2)** the authors might want to consider running simulations in which mutations are modelled as purely neutral, as the loss of genetic diversity in the strain A populations would be consistent with genetic drift acting more rapidly/powerfully on smaller populations.

*We thank the reviewer for their suggestions. We have now introduced in the main text six new references (line 139) which provide support for the use in our models of exponential distributed effects. We have also modified S3 Fig and its corresponding figure legend, which now includes a panel (A) that show the results for simulations using a fixed effect distribution or purely neutral.*

2. In response to our question about whether the M(t) statistic had been normalized by genome length, the authors added a line in the main text stating that they follow the approach of Good et al.—the study in which M(t) was originally developed, and in which the statistics was not normalized by genome length. However, we would like to point out that Good et al. applied this statistic to isogenic populations of E. coli that were allowed to evolve over time, and which therefore had the same genome length (at least they did at the beginning of the LTEE). This scenario differs from the study at hand, insofar as strains A and B1 had different genome lengths from the outset. That said, the authors may have decided not to normalize by genome length because the genome lengths of the two strains are similar enough to not qualitatively alter the M(t) results. If that is the case, we encourage the authors to say this explicitly, as it may allay readers’ concerns. It might also be advisable to communicate the actual size of each genome—if they are the same order of magnitude, it would further bolster the point that normalizing by genome size would not change the results.

*We acknowledge the Reviewer’s point, and changed the manuscript text accordingly in lines 212-215 and 240-244. Furthermore, we have now performed normalization of the M(t) by the genome size of each strain which did not change the results. This is now mentioned in lines 246-248.*

3. Typo on line 360 (“geneticdiversity” should be “genetic diversity”).

*The typo on line 360 has been corrected as suggested.*

**Reviewer #2**: This reviewer feels that the authors have adequately addressed all the questions I raised during my initial review. I would also like to note that I previously reviewed an earlier version of this manuscript when it was submitted to a different journal, and I find the current version to be significantly improved, both in terms of clarity and scientific content.

**Reviewer #3**: In the revision the authors have improved the presentation of the manuscript and clarified the potential sources of confusion regarding the interpretation of their results. In the process, they have addressed the questions raised in my previous review. I believe the manuscript will make a valuable contribution to the study of the role of HGT in bacterial evolution and have no further comments to add.

*We thank Reviewer 2 and Reviewer 3 for their helpful comments and suggestions, which have contributed to improving the manuscript.*
